# Olink proteomics reveals TNFRSF9 as a biomarker for abdominal aortic aneurysms

**DOI:** 10.1016/j.isci.2025.112828

**Published:** 2025-06-05

**Authors:** Runze Chang, Heng Wang, Chuanlong Lu, Jinshan Chen, Yaling Li, Maolin Qiao, Siqi Gao, Lizheng Li, Keyi Fan, Ruijing Zhang, Honglin Dong

**Affiliations:** 1 The Second Hospital of Shanxi Medical University, Taiyuan, Shanxi, China; 2 Centre for Transplant and Renal Research, Westmead Institute for Medical Research, The University of Sydney, Sydney, NSW 2145, Australia

**Keywords:** Cardiovascular medicine, Proteomics

## Abstract

Abdominal aortic aneurysm (AAA) is a serious cardiovascular disease associated with chronic inflammation. The purpose of this study was to use the Olink proteomics to reveal serum inflammatory markers in AAA. We examined the expression levels of 92 inflammation-related proteins in patients with AAA (*n* = 18) and healthy individuals (*n* = 10) using the Olink proximity extension assay (PEA) inflammatory plate. Olink proteomics identified 38 differential proteins. Combined analysis of Olink proteomics and GSE183464 showed interleukin-6 (IL-6) and tumor necrosis factor receptor superfamily member 9 (TNFRSF9) were upregulated at both gene and protein levels in AAA patients. The ELISA results were consistent with the Olink proteomics results, and the receiver operating characteristic (ROC) curve analysis revealed that the binding of TNFRSF9 and IL-6 has high diagnostic value (Olink AUC = 0.9056; ELISA AUC = 0.950). Subsequently, elevated TNFRSF9 expression in AAA was confirmed by animal models, suggesting that TNFRSF9 may serve as a potential biomarker for AAA.

## Highlights

• Olink proteomics reveals serum inflammation profiles in abdominal aortic aneurysm patients
• TNFRSF9 and IL-6 may serve as key markers with combined diagnostic value in abdominal aneurysm
• TNFRSF9 expression increases in serum and aortic tissue of abdominal aortic aneurysm mice
• Single-cell analysis shows TNFRSF9 is upregulated in T cells of abdominal aneurysm tissues

## Graphical abstract

**Figure:**
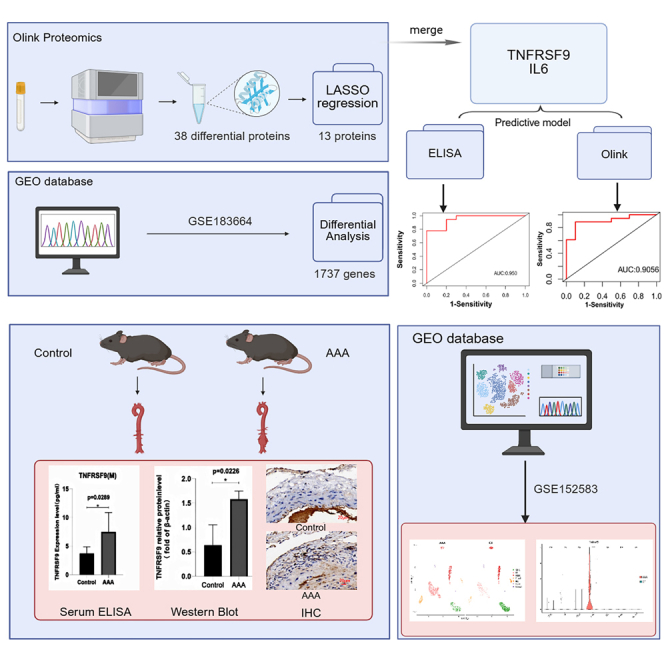

## Introduction

Abdominal aortic aneurysm (AAA) is a serious life-threatening cardiovascular disease that is characterized by irreversible dilatation of the abdominal aorta.^1^ AAA typically does not cause any symptoms in the early stages, and its diagnosis usually relies on medical imaging tests, such as ultrasound, computed tomography (CT), or MRI. Most often, AAA is discovered incidentally while performing a routine physical examination.^2^ Under normal conditions, the abdominal aorta measures approximately 20 mm in diameter. AAA is diagnosed when the diameter exceeds 30 mm.^3^ The most dangerous outcome of AAA is rupture, which has a death rate of 80%–90%.^4^^,^^5^ Currently, surgery is the main method for treating AAA.^6^ Due to the unknown mechanisms behind AAA, despite advancements in pharmacological therapy, there are currently no viable medications that can stop or slow the progression of the disease.^7^

AAA is a disease characterized by chronic inflammation.^8^ Increasing evidence indicates that inflammation is a key factor in the emergence and progression of AAA.^9^^,^^10^^,^^11^^,^^12^^,^^13^ There is infiltration of different inflammatory cells into the wall of the AAA. These inflammatory cells increase inflammation in AAAs by secreting several inflammatory agents, chemokines, and reactive oxygen species.^12^^,^^14^^,^^15^ To date, substantial evidence has demonstrated the involvement of multiple classical proinflammatory agents, including interleukin-1β (IL-1β), IL-18, and tumor necrosis fatcor alpha (TNF-α), in AAAs.^16^^,^^17^ Nevertheless, how peripheral immunity contributes to the inflammatory activation of AAAs is still unknown.

Proteomics is an established and widely used method for screening biomarkers for human diseases.^18^^,^^19^ Murakami Y. et al. collected serum samples from healthy individuals and patients with atherosclerotic thoracic aortic aneurysms for proteomic analysis. In their study, profilin 1 (PFN1) and complement factor D (CFD) were found to be two potential new biomarkers for the diagnosis of aortic aneurysms, and their combination could improve the diagnosis rate of aortic aneurysms.^20^ Olink proteomics is a novel high-throughput proteomic analysis method based on proximity extension assay (PEA) technology.^21^ The stability and reproducibility of Olink PEA technology has been demonstrated in multiple studies.^22^ Olink proteomics-based analysis of serum inflammatory biomarkers has been applied to a variety of diseases, such as adolescent depression, acute osteofascial compartment syndrome, and preeclampsia.^23^^,^^24^^,^^25^ In this study, the Olink PEA inflammation panel was utilized to identify differences in the expression of different inflammatory proteins between healthy individuals and patients with AAA. The aim of this study was to screen novel inflammatory biomarkers to facilitate further understanding of the role of inflammation in AAA and to potentially provide implications for clinical diagnosis.

## Results

### Baseline characteristics

Considering financial constraints and quality control limitations, a total of 10 healthy individuals and 18 patients with AAAs were included in this study. During the inclusion process, we ensured that the two groups were balanced in terms of gender and age to minimize the potential interference of these factors with the study results. Our results revealed no significant differences in age, gender, or smoking between the two groups of subjects (Table 1). However, there was a high incidence of hypertension in patients with AAA (*p* = 0.004), which is an important risk factor for the development of AAA. The red blood cell count was low in the AAA group (*p* = 0.017), which may be related to the lack of appetite and spiritual factors in patients with abdominal aortic aneurysms.

**Table 1:** Patient characteristics

	Level	AAA	Control	*p*
n	–	18	10	–
Maximum diameter (median [IQR])	–	4.91 [4.22, 5.18]	2.01 [1.95, 2.07]	<0.001
Age (median [IQR])	–	73.50 [69.00, 77.00]	67.00 [57.25,76.25]	0.269
Gender (%)	Male	16 (88.9)	5 (50.0)	0.069
	Female	2 (11.1)	5 (50.0)	
SBP (mean (SD))	–	131.83 (14.91)	134.40 (14.11)	0.66
DBP (mean (SD))	–	75.89 (12.29)	75.30 (10.46)	0.899
Height (mean (SD))	–	169.06 (7.08)	165.40 (3.84)	0.144
BMI (mean (SD))	–	24.53 (3.47)	26.31 (3.78)	0.219
Smoke (%)	No	5 (27.8)	6 (60.0)	0.204
	Yes	13 (72.2)	4 (40.0)	
Drunk (%)	No	12 (66.7)	5 (50.0)	0.644
	Yes	6 (33.3)	5 (50.0)	
Hypertension (%)	No	3 (16.7)	8 (80.0)	0.004
	Yes	15 (83.3)	2 (20.0)	
Diabetes (%)	No	14 (77.8)	7 (70.0)	1
	Yes	4 (22.2)	3 (30.0)	
Coronary artery disease (%)	No	13 (72.2)	9 (90.0)	0.537
	Yes	5 (27.8)	1 (10.0)	
GLU (median [IQR])	–	6.16 [4.91, 7.66]	5.45 [5.02, 7.42]	0.666
ESR (median [IQR])	–	11.50 [7.25, 20.00]	7.00 [6.00, 22.50]	0.335
TC (mean (SD))	–	4.50 (0.71)	5.04 (0.77)	0.071
TG (median [IQR])	–	1.30 [1.01, 1.81]	1.21 [1.06, 1.28]	0.565
HDL.C (median [IQR])	–	0.94 [0.89, 1.16]	1.30 [1.03, 1.40]	0.076
LDL.C (mean (SD))	–	2.42 (0.55)	2.63 (0.58)	0.357
WBC (median [IQR])	–	5.44 [4.78, 5.77]	5.82 [4.02, 6.15]	0.774
RBC (mean (SD))	–	4.29 (0.56)	4.88 (0.64)	0.017
PLT (mean (SD))	–	189.72 (38.23)	210.30 (46.85)	0.219
NEUT (mean (SD))	–	3.62 (0.95)	3.24 (1.03)	0.328
LYM (median [IQR])	–	1.56 [1.21, 1.71]	1.69 [1.08, 2.08]	0.598
MONO (mean (SD))	–	0.38 (0.09)	0.44 (0.14)	0.196
EO (median [IQR])	–	0.12 [0.08, 0.19]	0.08 [0.07, 0.11]	0.101
BASO (median [IQR])	–	0.03 [0.02, 0.03]	0.03 [0.03, 0.03]	0.406
NEUTR (mean (SD))	–	63.14 (5.11)	59.40 (7.99)	0.142
LYMR (mean (SD))	–	27.07 (4.95)	29.95 (7.44)	0.23
MONOR (mean (SD))	–	6.87 (1.68)	8.23 (2.07)	0.069
EOSR (median [IQR])	–	2.05 [1.80, 3.03]	1.45 [1.22, 1.67]	0.131
BASOR (mean (SD))	–	0.49 (0.24)	0.61 (0.23)	0.207
Antihypertensives (%)	No	6 (33.3)	8 (80.0)	0.055
	CCB	10 (55.6)	2 (20.0)	
	ACEI/ARB	2 (11.1)	0 (0.0)	
Hypoglycemic (%)	No	16 (88.9)	8 (80.0)	0.936
	Metformin	2 (11.1)	2 (20.0)	
Hypolipidemic (%)	No	12 (66.7)	9 (90.0)	0.362
	Atorvastatin	6 (33.3)	1 (10.0)	
Antiplatelet (%)	No	11 (61.1)	9 (90.0)	0.236
	Aspirin	7 (38.9)	1 (10.0)	

Abbreviations: AAA, abdominal aortic aneurysm; IQR: interquartile range; SD: standard deviation; SBP, systolic blood pressure; DBP, diastolic blood pressure; BMI, body mass index; GLU, glucose; TC, total cholesterol; TG, triglyceride; HDL.C, high-density lipoprotein; LDL.C, low density lipoprotein; WBC, white C, high-density lipoprotein; LDL.C, low density lipoprotein; WBC, white blood cell; RBC, red blood cell; NEUT, neutrophil granulocyte count; LYM, lymphocyte count; MONO, monocyte count; EO, eosinophil; BASO, basophil; NEUTR, neutrophil ratio; LYMR, lymphocyte ratio; MONOR, monocyte ratio; EOSR, eosinophil ratio; BASOR, basophil ratio; CCB, calcium channel blocker; ACEI, angiotensin-converting enzyme inhibitor; ARB, angiotensin receptor blocker.

### Comparison of Olink proteomics in AAAs and controls

**Figure 1:**
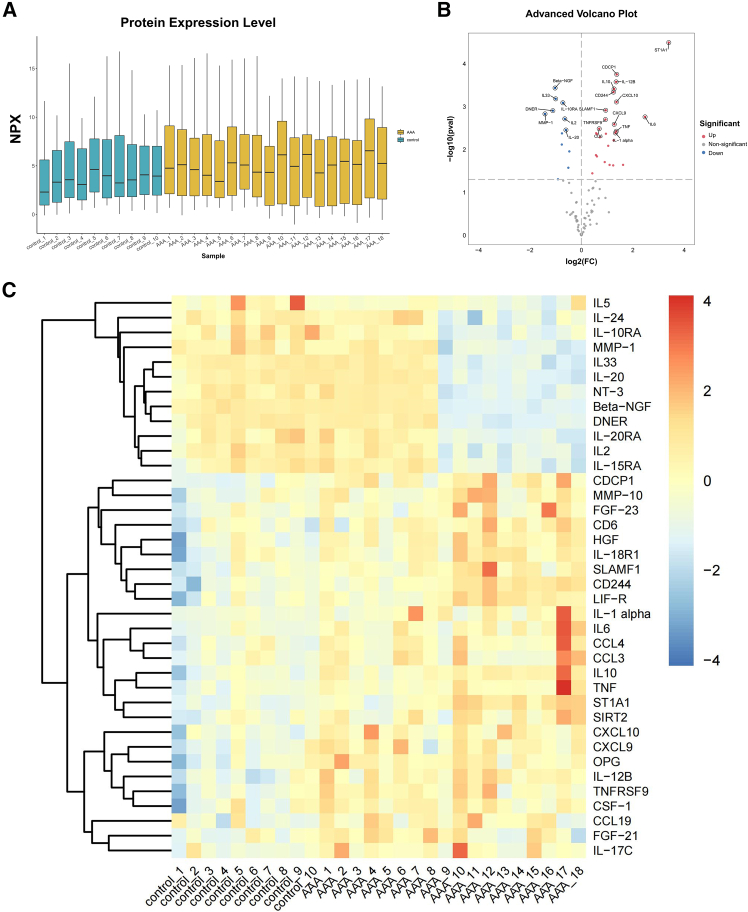
All DEPs of inflammation-related markers between the AAA (*n* = 18) and control groups (*n* = 10) (A) Protein expression in the AAA group and the control group. The *x* axis represents sample names, and the *y* axis shows NPX values. Different colors indicate different groups. Each boxplot displays five statistical measures (from top to bottom: maximum, upper quartile, median, lower quartile, and minimum. (B) Volcano plot of 92 inflammation-related proteins between the two groups. The horizontal axis in the figure represents the differential changes in protein expression (expressed as Log2(FC)), whereas the vertical axis represents the statistical significance of the differences in protein abundance (expressed as -Log2^10^(pval)). (C) Heatmap of DEPs between the two groups.

We compared the differences in the expression of 92 inflammation-related proteins in the serum of the AAA group and the control group by Olink proteomics analysis. Figure 1A shows the protein expression of all the samples. Compared with those in the control group, 38 differentially abundant proteins (DAPs) were identified in the AAA group; the expression of 26 proteins was upregulated, whereas the expression of 12 proteins was downregulated (Figure 1B). In addition, we presented the differential protein expression between the two groups and visualized the data using heatmaps (Figure 1C, Table S1).

**Figure 2:**
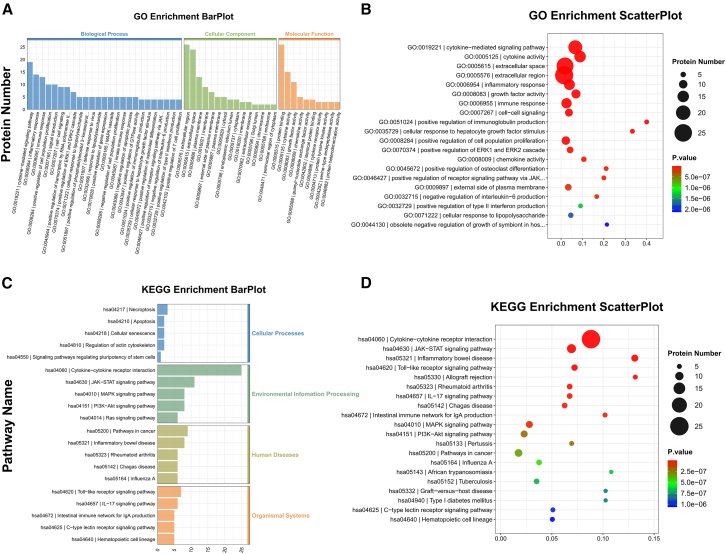
Gene ontology (GO) and Kyoto encyclopedia of genes and genomes (KEGG) enrichment analyses of differentially expressed inflammation-related proteins (A and B) Bar and bubble plots based on differential protein GO enrichment analysis. (C and D) Bar and bubble plots based on differential protein KEGG enrichment analysis.

We next analyzed the 38 DAPs for gene ontology (GO) and Kyoto encyclopedia of genes and genomes (KEGG) enrichment to investigate their potential impact. The results of the GO enrichment analysis revealed that the biological processes associated with these differentially expressed proteins were enriched mainly in the cytokine-mediated signaling pathway, inflammatory response, and immune response, which were enriched mainly in the extracellular space and extracellular region (Figures 2A and 2B). KEGG enrichment analysis revealed that these DAPs were enriched in the cytokine-cytokine receptor interaction, JAK-STAT signaling pathway, MAPK signaling pathway, and PI3K-Akt signaling pathway (Figures 2C and 2D).

### TNFRSF9 and IL-6 are characteristic genes of AAA

**Figure 3:**
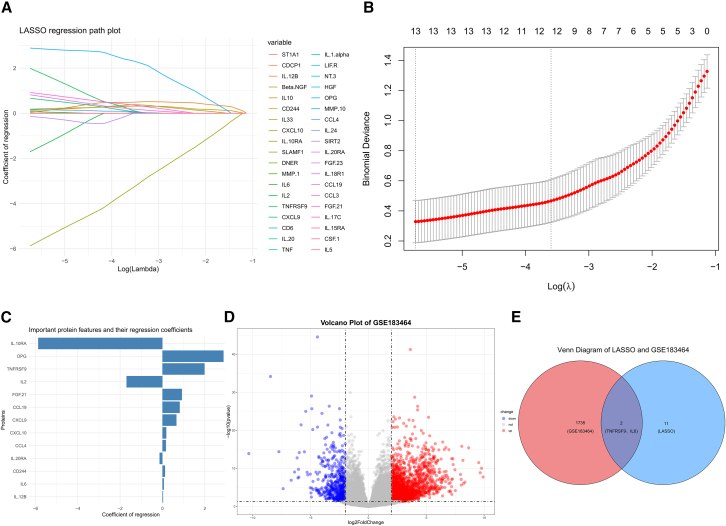
Screening for specific inflammation-related proteins between the two groups (A) LASSO regression path of differential inflammatory proteins. (B) Cross-validation curve of LASSO regression. (C) Bar plot of regression coefficients for important feature proteins. (D) Volcano plot of differentially expressed genes in the AAA group vs. control group in GSE183464. (E) Venn diagram of LASSO and GSE183464.

To better understand the characteristic genes associated with AAA, LASSO regression analysis was used to further screen the protein profiles associated with AAA. A total of 13 key characteristics proteins were identified (Figures 3A–3C). A comprehensive analysis of the publicly available GSE183464 dataset^26^ was subsequently conducted to investigate significant differences in mRNA expression between AAA patients (*n* = 7) and healthy individuals (*n* = 7). This analysis revealed that the expression of 1,737 genes significantly differed between the two groups (Figure 3D). Furthermore, by integrating the feature proteins identified through LASSO regression, we discovered that both interleukin-6 (IL-6) and tumor necrosis factor receptor superfamily member 9 (TNFRSF9) were upregulated at both the mRNA and protein levels (Figure 3E). These findings provide compelling evidence for the pivotal role of IL-6 and TNFRSF9 in AAA development.

**Figure 4:**
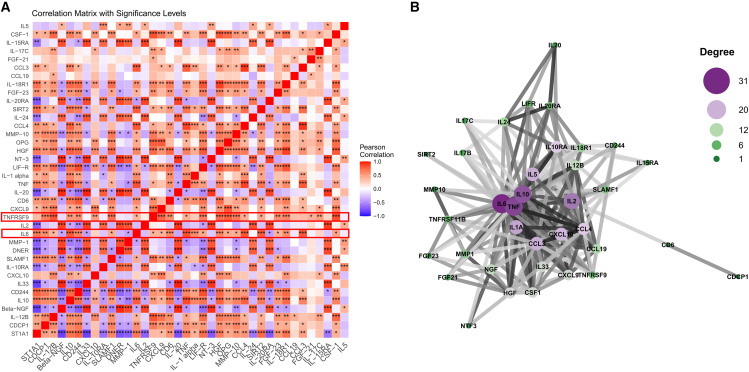
Correlation of TNFRSF9 and IL-6 with all DEPs (A) Correlation analysis between DEPs. (B) Visualization of the PPI network analysis of DEPs.

Furthermore, we performed a correlation analysis of the DAPs to investigate their interactions. The findings revealed significant correlations between IL-6 and TNFRSF9 and multiple proteins (Figure 4A). Subsequently, we utilized the STRING database to construct a protein-protein interaction (PPI) network in order to elucidate the connections among these DAPs (Figure 4B). Within the constructed PPI network, IL-6, TNF, and IL-10 were identified as core nodes because of their high connection scores. Notably, TNFRSF9 was observed to interact with seven proteins, including IL-2, CXCL10, CD244, IL-6, TNF, IL-10, and CCL3, all of which represented key nodes in the network (Table 2). This discovery underscores the potential pivotal role of TNFRSF9 in AAA development.

**Table 2:** PPI network analysis of TNFRSF9 and potential interacting proteins

Protein A	GeneA	Protein B	GeneB	Score threshold
9606.ENSP00000226730	IL2	9606.ENSP00000478699	TNFRSF9	739
9606.ENSP00000305651	CXCL10	9606.ENSP00000478699	TNFRSF9	425
9606.ENSP00000357012	CD244	9606.ENSP00000478699	TNFRSF9	504
9606.ENSP00000385675	IL6	9606.ENSP00000478699	TNFRSF9	553
9606.ENSP00000398698	TNF	9606.ENSP00000478699	TNFRSF9	710
9606.ENSP00000412237	IL10	9606.ENSP00000478699	TNFRSF9	622
9606.ENSP00000477908	CCL3	9606.ENSP00000478699	TNFRSF9	432

### TNFRSF9 combined with IL-6 has a better predictive value in AAA

**Figure 5:**
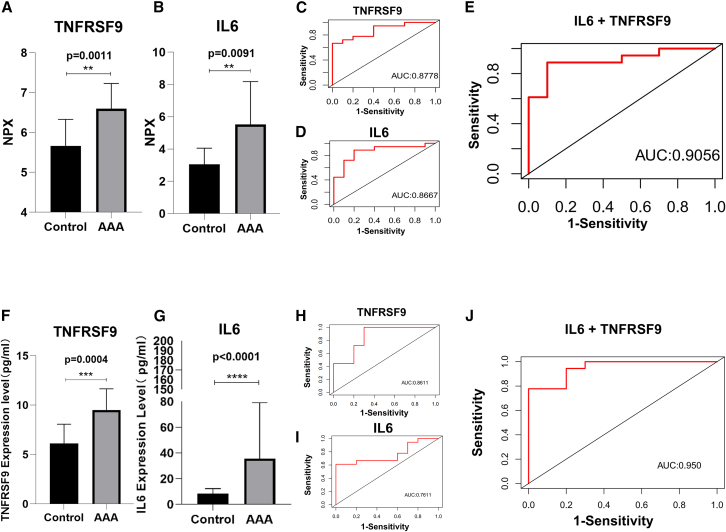
Predictive value of NPX values and ELISA values for IL-6 and TNFRSF9 (A) Olink data visualization of TNFRSF9. (B) Olink data visualization of IL-6. (C) Receiver operating characteristic (ROC) curve of TNFRSF9 concentrations based on Olink data between AAA and control groups. (D) ROC curve of IL-6 concentrations based on Olink data in the AAA and control groups. (E) ROC curve of IL-6+TNFRSF9 concentrations based on Olink data in the AAA and control groups. (F) ELISA data visualization of TNFRSF9. (G) ELISA data visualization of IL-6. (H) ROC curve of TNFRSF9 based on ELISA data in the AAA and control groups. (I) ROC curve of IL-6 concentrations based on ELISA data in the AAA and control groups. (J) ROC curve of IL-6+TNFRSF9 concentrations based on ELISA data in the AAA and control groups. Data are represented as mean ± SEMs. Statistical analysis between the two groups was performed using a t test. Significance levels are annotated as follows: ns = not significant, ∗ = *p* value < 0.05, ∗∗ = *p* value < 0.01, ∗∗∗ = *p* value < 0.001, ∗∗∗∗ = *p* value < 0.0001.

On the basis of the aforementioned results, we analyzed the diagnostic potential of IL-6 and TNFRSF9 in AAA. Figures 5A and 5B show the differences in the expression levels of IL-6 and TNFRSF9 between the two groups via Olink proteomics (Figures 5A and 5B). The receiver operating characteristic (ROC) curve analysis revealed an area under the curve (AUC) value of 0.8667 for IL-6 and 0.8778 for TNFRSF9, indicating their substantial diagnostic utility (Figures 5C and 5D). Furthermore, logistic regression-based ROC curve analysis incorporating both IL-6 and TNFRSF9 demonstrated an AUC value of 0.9056 (Figure 5E), suggesting that their combined use can enhance the diagnostic efficacy for AAA. The ELISA results revealed that the serum levels of IL-6 and TNFRSF9 were significantly greater in the AAA patients than in the controls (Figures 5F and 5G), which is consistent with the results of the Olink assay. ROC curve analysis revealed an AUC of 0.7611 for IL-6, 0.8611 for TNFRSF9 (Figures 5H and 5I), and 0.950 for the model constructed by logistic regression (Figure 5J), which also reflected good diagnostic value. These findings suggest that combining IL-6 and TNFRSF9 may be a new model for diagnosing AAA. However, validation of the logistic regression model using permutation testing revealed that the model’s performance was not stable (Figures S1A and S1B). This instability may be related to our small sample size, and further validation with a larger sample size may be needed in future studies. In addition, analysis of the correlation between the serum levels of TNFRSF9 and IL-6 and the maximum diameter of the aneurysm in AAA patients (Figure S2A–S2D) revealed no statistically significant association. This finding suggests that although elevated levels of TNFRSF9 and IL-6 may be associated with the development of AAA, they are not effective predictors of AAA disease severity.

### TNFRSF9 is highly expressed in AAA

**Figure 6:**
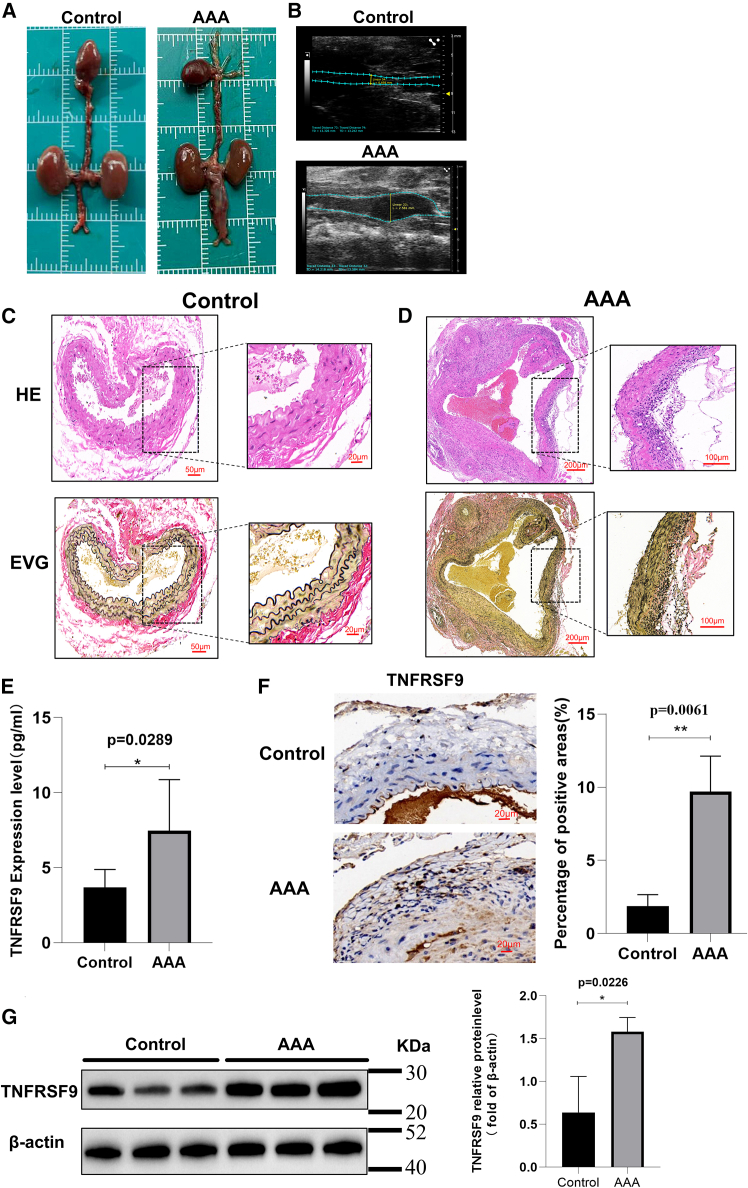
Validation of TNFRSF9 expression in AAA (A) Ordinary photographs of mouse aortic tissue. (B) Ultrasound images of the aortas of the mice before sacrifice. (C) Hematoxylin and eosin (H&E) staining of the abdominal aortas of the control and AAA groups. Scale bars: 50 μm (main image), 20 μm (inset). (D) Elastica van Gieson (EVG) staining of the abdominal aortas of the control and AAA groups. Scale bars: 200 μm (main image), 100 μm (inset). (E) Serum TNFRSF9 expression levels in the control and AAA groups (*n* = 6). (F) Comparison of TNFRSF9 expression between the two groups by immunohistochemistry. Scale bars: 20 μm. (G) Western blot analysis of TNFRSF9 protein and β-actin proteins. Data are represented as mean ± SEM. Statistical analysis between the two groups was performed using a t test. Significance levels are annotated as follows: ns = not significant, ∗ = *p* value < 0.05, ∗∗ = *p* value < 0.01, ∗∗∗ = *p* value < 0.001, ∗∗∗∗ = *p* value < 0.0001.

**Figure 7:**
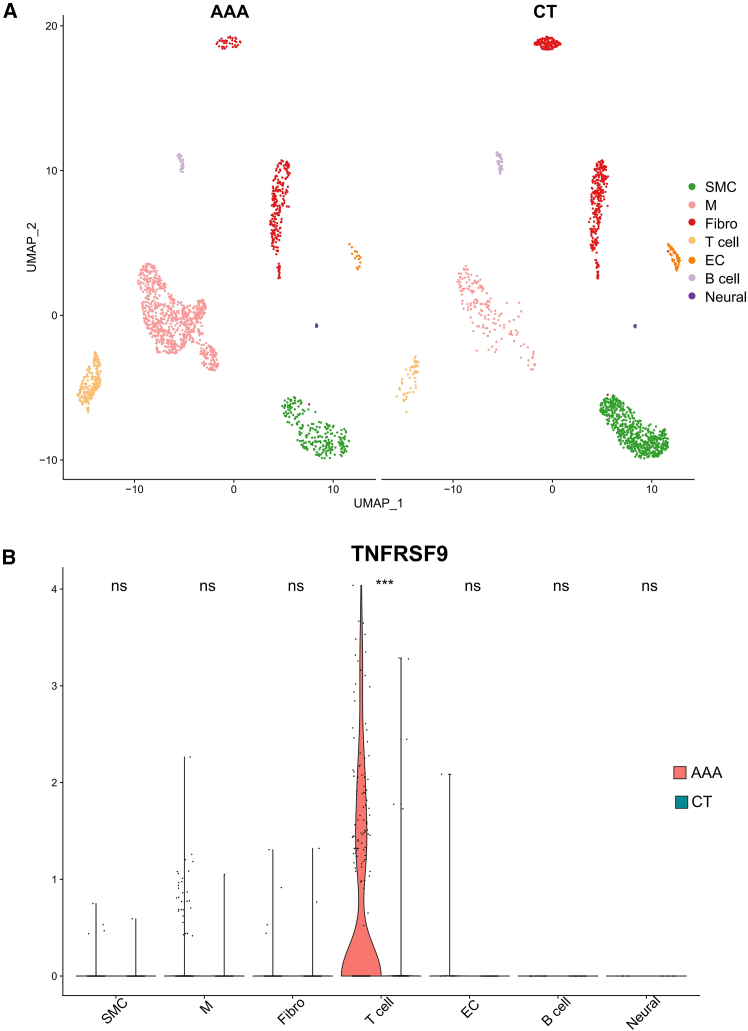
Single-cell data analysis of AAA (A) Uniform manifold approximation and projection (UMAP) images of single cells isolated from the abdominal aortas of control and AAA mice. (B) Tnfrsf9 expression in the T cells of AAA mice. Data are represented as mean ± SEMs. Significance levels are annotated as follows: ns = not significant, ∗ = *p* value < 0.05, ∗∗ = *p* value < 0.01, ∗∗∗ = *p* value < 0.001, ∗∗∗∗ = *p* value < 0.0001.

These results were validated using an animal model of AAA established in C57BL/6 mice. Ultrasound imaging and original photography verified the success of our model construction (Figures 6A and 6B), and H&E staining showed a significant increase in the infiltration of inflammatory cells into the vessel wall in the AAA group (Figure 6C). Moreover, elastic fibers in the AAA group were significantly damaged, as revealed by Elastica van Gieson (EVG) staining (Figure 6D). Various studies have demonstrated that IL-6, a typical inflammatory factor, has significantly elevated expression levels in AAA and plays a key role in the development of AAA. However, the role and expression levels of TNFRSF9 in AAA have not been explored in depth in any relevant studies. First, we examined TNFRSF9 levels in mouse serum by ELISA and found that the serum TNFRSF9 levels in AAA mice were significantly greater than those in control mice (Figure 6E), which is consistent with findings in clinical patients. To further explore its expression in diseased tissues, the abdominal aortic tissues of the mice were immunohistochemically stained. The results of the immunohistochemical staining demonstrated that, in the AAA group, the proportion of areas positive for TNFRSF9 was significantly greater than that in the control group (Figure 6F). In addition, we performed western blot analysis of aortic tissues from the mice, which revealed that the expression level of TNFRSF9 was significantly greater in the aortic tissues of the AAA mice than those of the control mice (Figure 6G). After the elevated levels of TNFRSF9 expression in aortic tissues were confirmed, we analyzed the publicly available AAA single-cell dataset GSE152583^27^ and found that TNFRSF9 was predominantly enriched in T cell subsets in AAA tissues (Figures 7A and 7B). This finding implies that the high expression of TNFRSF9 may affect the immune function of T cells, which in turn contributes to the development of AAA. Taken together, we conclude from these results that TNFRSF9 is a potential biomarker of AAA.

## Discussion

Abdominal aortic aneurysm is a cardiovascular disease that poses a serious risk to the human life.^3^ It has a complex pathomechanism, and it is believed that the inflammatory response plays a key role.^11^^,^^28^ In this study, we identified potential inflammatory biomarkers in patients with AAAs by Olink proteomics, aiming to provide directions for further research on AAA as well as ideas for the clinical diagnosis of this disease.

We first compared the expression of inflammation-related proteins in plasma samples from AAA patients and healthy individuals using Olink proteomics and identified 38 differentially expressed inflammation-associated proteins; the expression of 26 proteins was upregulated, whereas the expression of 12 proteins was downregulated. Among the 38 DAPs, the interleukin family dominated, including IL-6, IL-2, and IL-5, members of the CXC chemokine family, such as CXCL10 and CXCL9, and members of the CC chemokine family, CCL3 and CCL4, also differed. These inflammatory factors may play a key role in the development and progression of disease by regulating the inflammatory response and immunomodulatory processes in AAA. In addition, the expression of some tumor-related inflammatory factors, such as CDCP1 and TNFRSF9, are upregulated in AAA. Notably, a study of cardiovascular disease using Mendelian randomization revealed that CDCP1 is an important risk factor for coronary artery disease.^29^ In addition, differences in the expression of immune cell surface markers, such as CD244, SLAMF1, and CD6 implied that immune cell dysfunction may play an important role in the inflammatory response to AAA. In conclusion, dysregulation of these inflammatory proteins plays an essential role in the disease of AAA. GO and KEGG enrichment analyses revealed that these proteins were located mainly in the extracellular space and enriched in pathways, such as the cytokine-cytokine receptor interaction pathway, the JAK-STAT signaling pathway, the MAPK signaling pathway, and the PI3K-Akt signaling pathway. Using LASSO regression analysis, we successfully identified 13 characteristic proteins, including IL-12B, CD244, CXCL10, IL-10RA, IL-6, IL-2, TNFRSF9, CXCL9, OPG, CCL4, IL-20RA, CCL19, and FGF21. GSE183464 is a dataset in the Gene Expression Omnibus (GEO) public database that contains mRNA sequence information of 7 AAA samples and 7 control samples. In this dataset, we identified 1,737 genes that were differentially expressed in AAA samples. Further analysis revealed that IL-6 and TNFRSF9 were upregulated at both the gene and protein levels, suggesting that they may play crucial roles in the pathogenesis of AAA. Correlation analysis revealed a significant association of IL-6 and TNFRSF9 with the expression of most of the differentially expressed proteins. PPI network analysis further revealed that IL-6, TNF, and IL-10 are core proteins involved in the inflammatory response and also revealed that TNFRSF9 may interact with seven other inflammation-related proteins. IL-6, a classic proinflammatory cytokine, is produced and released by a variety of cell types, including monocytes and macrophages. It has been shown to play a key role in inflammatory responses, tumor development, and a variety of other disease processes.^30^ Previous studies have shown that circulating levels of IL-6 correlate with the progression of abdominal aortic aneurysms.^31^^,^^32^ Furthermore, several studies have shown that blocking high IL-6 expression significantly inhibits aortic dilation.^33^^,^^34^^,^^35^ TNFRSF9, also known as CD137, belongs to the TNF receptor superfamily. It is expressed mostly on the surface of activated T cells and contributes to their proliferation and survival. TNFRSF9 is also expressed in small amounts on other cell types, such as endothelial cells, macrophages, and monocytes.^36^ TNFRSF9 has received attention in the field of tumor immunology. Studies have shown that the activity of natural killer (NK) cells and T cells can be modulated by targeting TNFRSF9. Currently, monoclonal antibodies against TNFRSF9 have been extensively tested in several tumor-related clinical trials.^37^ Recent studies have revealed the important role of TNFRSF9 in cardiovascular disease. Following acute myocardial infarction, the heart often undergoes myocardial fibrosis. Studies have shown that inhibition of the TNFRSF9 signaling pathway helps attenuate pathological cardiac remodeling and has a positive effect on improving cardiac function after myocardial infarction. Conversely, activation of the TNFRSF9 signaling pathway may lead to necrosis of cardiomyocytes, thereby exacerbating myocardial ischemia-reperfusion injury.^38^^,^^39^ Furthermore, in patients with acute coronary syndrome (ACS), the proportion of CD8^+^CD69^+^CD137^+^ cells was significantly increased in peripheral blood mononuclear cells (PBMC) after activation by the autoantigen LL-37. This phenomenon implies that T cells expressing TNFRSF9 play an important role in immune signaling in ACS.^40^ In the pathological process of atherosclerosis, the activation of TNFRSF9 is strongly correlated with the exacerbation of plaque formation and the vascular calcification process.^41^^,^^42^ Therefore, TNFRSF9 is considered a potential biomarker of atherosclerosis.^43^ When endothelial cells activate the TNFRSF9 signaling pathway in response to inflammatory stimuli, this process is able to reduce the translocation of TET2 proteins in exosomes released by endothelial cells to vascular smooth muscle cells (VSMCs). This mechanism may inhibit the phenotypic switching of VSMCs, which in turn promotes the development of atherosclerotic plaques.^44^ Activation of TNFRSF9 signaling on the macrophage surface modulates the STAT6/PPARδ signaling pathway, which induces the polarization of macrophages in atherosclerosis toward the M2 type.^45^ These studies revealed an important role for TNFRSF9 in the development of cardiovascular disease. Nevertheless, the specific role of TNFRSF9 in AAAs remains to be further elucidated. Both Olink proteomics and ELISA revealed significantly elevated levels of TNFRSF9 expression in the peripheral circulation of AAA patients. ROC analysis of the logistic model constructed by combining TNFRSF9 and IL-6 showed high potential for diagnosing AAA (Olink AUC = 0.9056, ELISA AUC = 0.950). In addition, the high expression of TNFRSF9 in AAA tissues was further confirmed by immunohistochemistry and Western blot analysis of the animal model. Furthermore, analysis of the GSE152583 single-cell dataset revealed that TNFRSF9 was highly expressed in the T cells of AAA model mice. This phenomenon may indicate that TNFRSF9 promotes AAA-associated inflammatory responses by regulating T cell function, which in turn triggers an immune imbalance. These findings may indicate that TNFRSF9 is a potential AAA biomarker.

In summary, this study analyzed serum samples from healthy individuals and AAA serum samples using Olink proteomics and successfully identified 38 DAPs. Through combined analysis with the public dataset GSE183464, we specifically focused on two proteins, IL-6 and TNFRSF9, as biomarkers of AAA. Next, we applied ROC curve analysis to evaluate the predictive efficacy of these biomarkers as diagnostic indicators. In addition, we validated these results by ELISA and an animal model. Despite the limited sample size of this study, the results still suggest that TNFRSF9 may serve as a new biomarker for AAA and provide a valuable reference for future studies.

### Limitations of the study

There are several limitations of our study. First, our clinical sample size was small, and large cohort studies are still needed to validate our results. Second, populations with other cardiovascular diseases, such as myocardial infarction, coronary artery disease, atherosclerosis, and stroke, were not included, which could help further determine the specificity of elevated TNFRSF9 in AAA. Third, we used publicly available tissue mRNA expression data to assist in the selection of differentially expressed serum proteins identified via Olink proteomics analysis. This strategy may have led to the omission of some potential biomarkers. Furthermore, we did not perform further grading of the AAAs, which may have led to the poor adaptability of our results. Even so, we identified TNFRSF9 as a target worthy of further study and identified new biomarkers for clinical diagnosis through proteomic analysis and ELISA validation of AAA patients and healthy subjects.

## Resource availability

### Lead contact

For additional information or requests for resources and reagents, please contact the lead contact, Honglin Dong (honglindong@sxmu.edu.cn).

### Materials availability

No new unique reagents or materials were generated in this study.

### Data and code availability

• Data: The Olink proteomics sequencing data have been uploaded in Data S1. Publicly available datasets were analyzed in this study. These data can be found here: GSE183464; GSE152583. The sources for the datasets are listed in the key resources table.
• Code: This study does not report original code.
• Additional information requests: Any additional information necessary to reanalyze the data used in this study is available from the lead contact upon request.

## Acknowledgments

This work received the support from the Regional Cooperation Program of Shanxi Province, China (grant no. 202204041101038); the Leading Talent Team Building Program of Shanxi Province, China (grant no. 202204051002010); Construction and Demonstration of Molecular Diagnosis and Treatment Platform for Vascular Diseases in Shanxi Province, China (grant no. SCP-2023-17); Translational Medicine Engineering Research Center for Vascular Diseases of Shanxi Province, China (grant no. 2022017); The Shanxi Provincial Science and Technology Department Centralized Guided Local Projects (grant no. YDZJSX2021C026); and The Shanxi Province Graduate Education Innovation Program (grant no. 2024SJ169).

## Author contributions

Conceptualization, H.D. and R.Z.; data curation, R.C., H.W., J.C., Y.L., S.G., L.L., and K.F.; formal analysis, R.C., C.L., and M.Q.; writing – original draft preparation, R.C.; writing - review and editing, H.D. and R.Z..

## Declaration of interests

The authors declare no competing interests.

## STAR★Methods

### Key resources table

**Table:** 

REAGENT or RESOURCE	SOURCE	IDENTIFIER
**Antibodies**		

CD137 Mouse Monoclonal Antibody [2G1] (M1701-11)	HUABIO	Cat# M1701-11; RRID: AB_3073166
Anti-beta Actin antibody	Abcam	Cat# ab8227; RRID: AB_2305186

**Biological samples**		

Human serum	Homo sapiens	Ethics Committee of the Second Hospital of Shanxi Medical University approved the study (2024) YX No. (016)
Mouse samples	Mus musculus	Animal Ethics Committee of the Second Hospital of Shanxi Medical University (DW2023048)

**Chemicals, peptides, and recombinant proteins**		

Elastase, From Porcine Pancreas	Shanghai yuanye Biotechnology	CAS#39445-21-1
β-Aminopropionitrile	MedChem Express	CAS#151-18-8

**Critical commercial assays**		

Human IL-6 ELISA	Wuhan Servicebio Biotechnology	GEH0001
Human TNFRSF9 ELISA	Quanzhou Ruixin Biotechnology	RX106062H
Mouse TNFRSF9 ELISA	Quanzhou Ruixin Biotechnology	RX2DW2003666

**Deposited data**		

RNA-seq data	Zhang et al.^26^	GSE183464
single-cell RNA sequencing data	Zhao et al.^27^	GSE152583
Olink proteomics sequencing data	This paper	Data S1

**Experimental models: Organisms/strains**		

Mouse: C57BL/6J	Nanjing Junke Bio	JKM202

**Software and algorithms**		

GraphPad Prism	GraphPad Software	Version 8.0
R	The R Project for Statistical Computing	Version 4.2.2
ImageJ	ImageJ software	Version 1.54a
Vevo 2100 platform	Visual Sonics	https://www.visualsonics.com/

### Experimental model and study participant details

#### Mouse models of abdominal aortic aneurysm

All animal experiments were conducted in accordance with institutional guidelines and received approval from the Animal Ethics Committee of the Second Hospital of Shanxi Medical University. (DW2023048) Male C57BL/6 mice (6−8 weeks old; Nanjing Junke Bio Co., Ltd., China) were randomly divided into AAA (n=6) and control groups (n=6). After a midline abdominal incision was made to expose the abdominal aorta, the abdominal aorta below the kidney was topically treated for 20 minutes with a gel sponge containing porcine pancreatic elastase (100 mg/ml)(CAS#39445-21-1, Shanghai yuanye Biotechnology Co., Ltd., China). After treatment, the gel sponge was removed, and the abdominal cavity was thoroughly rinsed with 0.9% saline, followed by suturing. As a control, a gel sponge soaked in 0.9% saline was used in another group of mice. After surgery, the mice were placed on insulated heating pads to promote awakening and recovery. The water for the AAA group included 0.15% (v/v) β-aminopropionitrile (BAPN)(HY-Y1750, MedChem Express). The abdominal aortas of the mice were removed after 4 weeks.

#### Human serum sample collection

This study involving human subjects was performed in accordance with the principles of the Declaration of Helsinki. The Ethics Committee of the Second Hospital of Shanxi Medical University approved the study (2024) YX No. (016). Informed consent was obtained from patients with abdominal aortic aneurysms and healthy individuals. Owing to the complexity of obtaining clinical samples and financial constraints, we collected serum samples from 10 healthy individuals and 18 patients with abdominal aortic aneurysms.

The inclusion criteria for patients with AAA were as follows:1) aged greater than 18 years and less than 85 years; and 2) the maximum diameter of the abdominal aorta was ≥30 mm, or the maximum diameter of the aneurysm was more than 50% of the diameter of the adjacent normal aorta.

The exclusion criteria were as follows: 1) the presence of malignancy; 2) a history of comorbidities that may affect inflammation, such as infections and autoimmune diseases; and 3) refusal to provide informed consent.

Healthy individuals were matched in the outpatient clinic on the basis of the sex and age of the AAA patients to eliminate potential confounding effects, and informed consent was obtained from the individuals. We collected 5 ml of peripheral venous blood from 10 healthy individuals and 18 patients with AAA into tubes. The serum was then extracted, centrifuged at 3000 rpm for 10 min, and stored at - 80°C for further analysis.

### Method details

#### Olink proteomics analysis

Proteins were measured using the Olink® target 96 panel (Olink Proteomics AB, Uppsala, Sweden) according to the manufacturer’s instructions. The proximity extension assay (PEA) technology used for the Olink protocol has been well described and enables 92 analytes to be analyzed simultaneously, using 1 μL of each sample. In brief, pairs of oligonucleotide-labeled antibody probes bind to their targeted protein, and if the two probes are brought in close proximity the oligonucleotides hybridize in a pairwise manner. The addition of a DNA polymerase leads to a proximity-dependent DNA polymerization event, generating a unique PCR target sequence. The resulting DNA sequence is subsequently detected and quantified using a microfluidic real-time PCR instrument for normalization (Signature Q100, LC-Bio Technology Co., Ltd., Hangzhou, China). Data are then quality controlled and using an internal extension control and an interplate control are used to adjust for intra- and inter-run variation. The final assay read-out is presented in normalized protein expression (NPX) values, which is an arbitrary unit on a log2 scale where a high value corresponds to increased protein expression. The Benjamini‒Hochberg method was used for multiple correction during the analysis to reduce the false-positive rate and ensure the robustness of the results. All assay validation data (detection limits, intra- and interassay precision data, etc.) are available on the manufacturer’s website (www.olink.com).

#### Lasso regression analysis

Using LASSO regression, we further selected feature proteins from the differentially abundant proteins (DAPs) screened by Olink proteomics. LASSO regression applies L1 regularization to compress the regression coefficients, effectively performing variable selection, reducing model complexity, and retaining key features that significantly contribute to the outcome. To assess the stability and reliability of the model, we employed leave-one-out cross-validation (LOOCV). This method allowed for internal validation of the model, ensuring the consistency and robustness of the selected feature proteins across different datasets.

#### Bioinformatics analysis

Based on the DAPs screened by Olink proteomics, GO enrichment analysis and KEGG enrichment analysis were performed. The selected DAPs were mapped to the GO database, and the enrichment of each GO term was calculated. The significance was assessed using hypergeometric testing, while the Benjamini-Hochberg method was applied for multiple testing correction to control the false discovery rate (FDR). The RNA sequencing (RNA-seq) and single-cell RNA sequencing (scRNA-seq) data used in this study were both sourced from the Gene Expression Omnibus (GEO, www.ncbi.nlm.nih.gov/geo) database.

#### RNA-seq data analysis

We obtained RNA-seq data from the GSE183464 dataset, which includes 7 AAA and 7 Control samples, and conducted an in-depth analysis. After performing quality control on the raw sequencing data using FastQC, the data was aligned to the reference genome using HISAT2, and gene expression levels were quantified using featureCounts. Subsequently, differential expression analysis was performed using DESeq2 to identify significantly differentially expressed genes (Log2FC > 1 and p < 0.05).

#### Single-cell RNA-seq data analysis

The mouse single-cell RNA sequencing data were sourced from GSE152583. In this dataset, the control group consists of mice treated with heat-inactivated elastase for 14 days, while the AAA group consists of mice treated with elastase for 7 days, aiming to explore the transcriptional changes of different cell populations in the AAA model. Data preprocessing included the removal of low-quality cells, normalization, principal component analysis (PCA), and dimensionality reduction using UMAP/t-SNE. Cell clustering was performed using the FindClusters algorithm, and cell types were annotated based on known marker genes. Finally, we performed a comparative analysis of Tnfrsf9 expression between the two groups.

#### ELISA validation

Human tumor necrosis factor receptor superfamily member 9 (TNFRSF9) (RX106062H, Quanzhou Ruixin Biotechnology Co., Ltd., China) and interleukin 6 (IL-6) ELISA kits were used. (GEH0001, Wuhan Servicebio Biotechnology Co., Ltd., China) Serum samples from 18 patients with abdominal aortic aneurysms and 10 healthy individuals were analyzed by ELISA. Mouse TNFRSF9 ELISA kits were used. (RX2DW2003666, Quanzhou Ruixin Biotechnology Co., Ltd., China). Serum samples from 6 mice from the AAA group and 6 mice from the control group were analyzed by ELISA. Determination and analysis were performed according to the manufacturer’s protocol.

#### Ultrasound imaging

The Vevo 2100 platform (Visual Sonics, Toronto, CA, USA) was used to monitor the diameter of the abdominal aorta in themice using ultrasound.

#### Histopathological experiment

Mouse abdominal aortic tissues were first rinsed with cold PBS and then fixed with 4% paraformaldehyde for more than 12 hours. Paraffin-embedded sections were stained with hematoxylin-eosin(HE) and Elastica van Gieson (EVG) for histologic examination.

#### Western blot analysis

After extracting proteins from the abdominal aortic tissues of the mice, the proteins were first separated by gel electrophoresis and then transferred to membranes, followed by incubation with an antibody to specifically bind the target proteins. Through this process, the expression of TNFRSF9(HUABIO, M1701-11) and β-actin (Abcam, ab8227) was determined in both sample groups. Semiquantitative analysis of the Western blot bands was performed via ImageJ.

#### Immunohistochemical staining

After aortic tissue samples were obtained from the mice, they were first fixed and subsequently embedded in paraffin. After embedding, the tissue was cut into thin slices for immunohistochemical (IHC) staining for TNFRSF9 (HUABIO, M1701-11) TNFRSF9-positive areas were analyzed via ImageJ.

### Quantification and statistical analysis

We used R software (version 4.2) with GraphPad Prism 8.0 for statistical and bioinformatics analyses. For continuous variables, t tests were used to express data as the mean ± SEM if the measurements met the normality criterion, and the Mann‒Whitney U test was used to perform statistical analyses between groups if the normality criterion was not met. The chi-square test was used for count data. Characteristic proteins were screened via LASSO regression. Pearson correlation analysis revealed correlations between the expression of differential proteins. We plotted ROC curves for IL6 and TNFRSF9. A logistic regression model was used to calculate the ROC curve. The stability of the logistic model was subsequently validated through permutation testing. In addition, Spearman correlation analysis revealed a correlation between the maximum diameter and TNFRSF9 or IL6. The P value cutoff was selected as 0.05.

## Supplemental information

Document S1. Figures S1, S2, and Table S1

Data S1. Olink proteomics sequencing data
