# Supplementary material for: Olink proteomics reveals TNFRSF9 as a biomarker for abdominal aortic aneurysms
Source: iScience. 2025 Jun 5;28(7):112828. doi: 10.1016/j.isci.2025.112828 (PMC12209979; doi:10.1016/j.isci.2025.112828)
Supplement: Document S1. Figures S1, S2, and Table S1 [file mmc1.pdf]

iScience, Volume *28*

Supplemental information

Olink proteomics reveals TNFRSF9 as a biomarker for abdominal aortic aneurysms

Runze Chang, Heng Wang, Chuanlong Lu, Jinshan Chen, Yaling Li, Maolin Qiao, Siqi Gao, Lizheng Li, Keyi Fan, Ruijing Zhang, and Honglin Dong

Supplement Tables

	AAA	Control	p_val	Adjusted_pval
**N**	18	10		
**ST1A1 (mean (SD))**	7.14 (2.39)	3.74 (1.18)	<0.001	0.003
**CDCP1 (mean (SD))**	5.17 (1.05)	3.80 (0.61)	<0.001	0.007
**IL.12B (mean (SD))**	7.09 (0.72)	5.74 (0.77)	<0.001	0.007
**Beta.NGF (mean (SD))**	0.96 (0.97)	1.99 (0.24)	<0.001	0.007
**IL10 (mean (SD))**	3.70 (0.85)	2.43 (0.72)	<0.001	0.007
**CD244 (mean (SD))**	6.24 (0.69)	4.99 (0.76)	<0.001	0.007
**IL33 (mean (SD))**	0.58 (1.00)	1.58 (0.26)	0.001	0.008
**CXCL10 (mean (SD))**	9.24 (0.91)	7.88 (0.85)	0.001	0.008
**IL.10RA (mean (SD))**	0.54 (0.41)	1.26 (0.47)	0.001	0.008
**SLAMF1 (mean (SD))**	2.81 (0.75)	1.87 (0.58)	0.001	0.010
**DNER (mean (SD))**	8.00 (1.14)	9.11 (0.46)	0.001	0.010
**MMP.1 (mean (SD))**	14.56 (1.32)	15.97 (0.79)	0.001	0.011
**IL6 (mean (SD))**	5.52 (2.65)	3.05 (1.01)	0.002	0.012
**IL2 (mean (SD))**	0.47 (0.66)	1.12 (0.34)	0.002	0.012
**TNFRSF9 (mean (SD))**	6.60 (0.63)	5.66 (0.67)	0.002	0.012
**CXCL9 (mean (SD))**	8.14 (1.01)	6.86 (0.91)	0.003	0.015
**CD6 (mean (SD))**	5.60 (0.53)	4.91 (0.52)	0.003	0.017
**IL.20 (mean (SD))**	0.46 (0.71)	1.06 (0.25)	0.004	0.017
**TNF (mean (SD))**	4.74 (1.55)	3.38 (0.69)	0.004	0.017
**IL.1.alpha (mean (SD))**	1.64 (1.66)	0.32 (0.35)	0.004	0.017
**LIF.R (mean (SD))**	3.85 (0.36)	3.28 (0.47)	0.004	0.017
**NT.3 (mean (SD))**	1.98 (0.87)	2.73 (0.40)	0.004	0.017
**HGF (mean (SD))**	9.69 (0.51)	8.74 (0.79)	0.004	0.017
**OPG (mean (SD))**	10.68 (0.35)	10.07 (0.50)	0.004	0.017
**MMP.10 (mean (SD))**	9.02 (0.65)	8.31 (0.54)	0.005	0.019
**CCL4 (mean (SD))**	8.62 (1.28)	7.37 (0.91)	0.006	0.022
**IL.24 (mean (SD))**	0.89 (0.96)	1.65 (0.43)	0.008	0.026
**SIRT2 (mean (SD))**	3.27 (1.13)	2.22 (0.77)	0.008	0.027
**IL.20RA (mean (SD))**	0.81 (0.51)	1.28 (0.39)	0.011	0.035
**FGF.23 (mean (SD))**	1.40 (0.72)	0.84 (0.41)	0.014	0.041
**IL.18R1 (mean (SD))**	7.64 (0.39)	7.04 (0.61)	0.014	0.043
**CCL19 (mean (SD))**	11.33 (0.93)	10.42 (0.89)	0.020	0.056
**CCL3 (mean (SD))**	8.74 (2.33)	7.12 (1.21)	0.023	0.062
**FGF.21 (mean (SD))**	6.62 (1.40)	5.52 (0.97)	0.023	0.062
**IL.17C (mean (SD))**	3.45 (1.68)	2.22 (1.03)	0.023	0.062
**IL.15RA (mean (SD))**	1.19 (0.67)	1.70 (0.46)	0.027	0.068
**CSF.1 (mean (SD))**	10.16 (0.27)	9.74 (0.53)	0.036	0.089
**IL5 (mean (SD))**	0.61 (0.57)	1.52 (1.24)	0.042	0.120

Supplemental Table 1. Expression levels of 38 differentially expressed inflammatory proteins. SD, standard deviation.

Supplement Figures

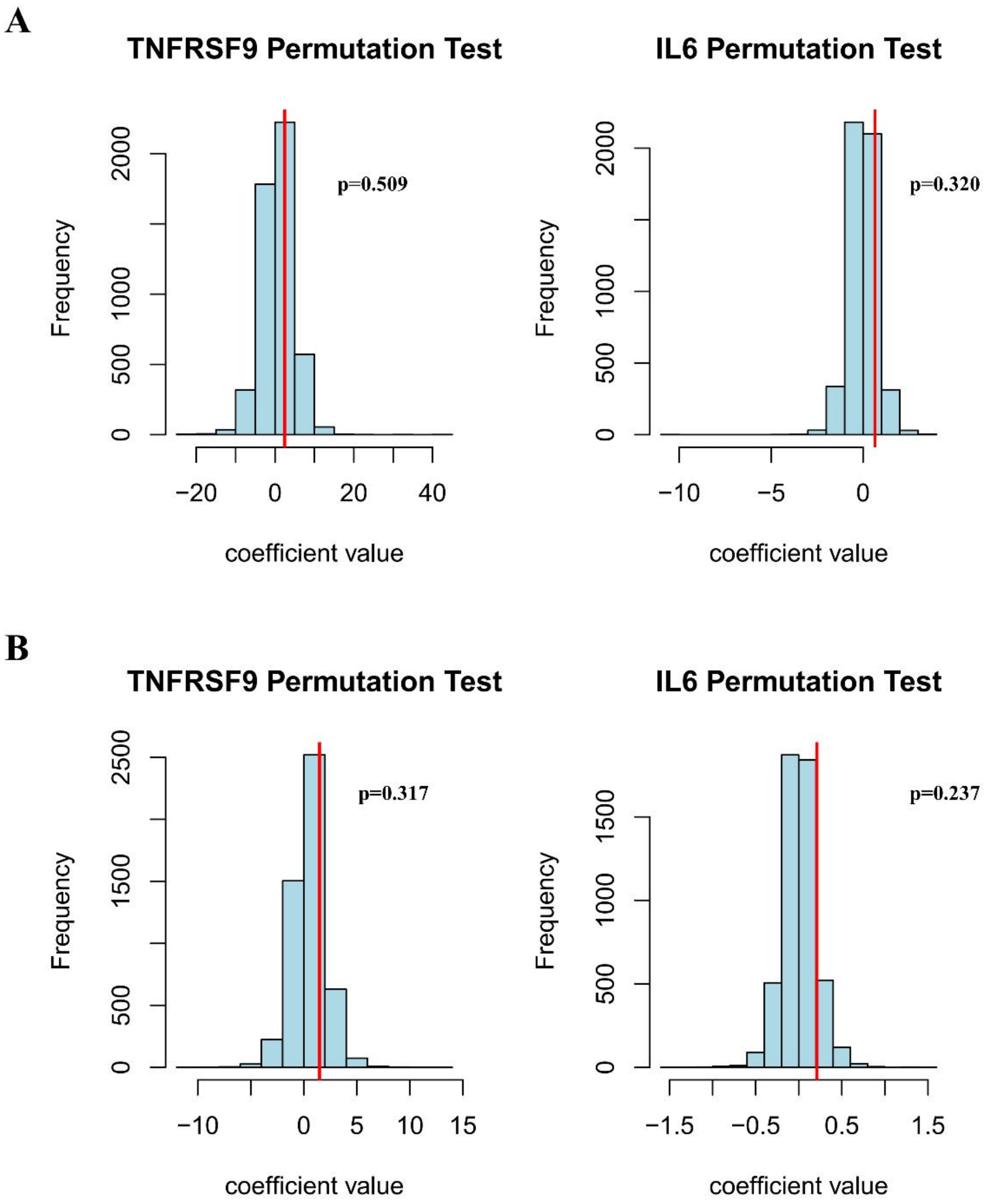

Supplemental Figure 1. Permutation test for the logistic regression model. (A) Permutation test for the logistic regression model constructed on the basis of NPX values. (B) Permutation test for the logistic regression model constructed on the basis of ELISA values.

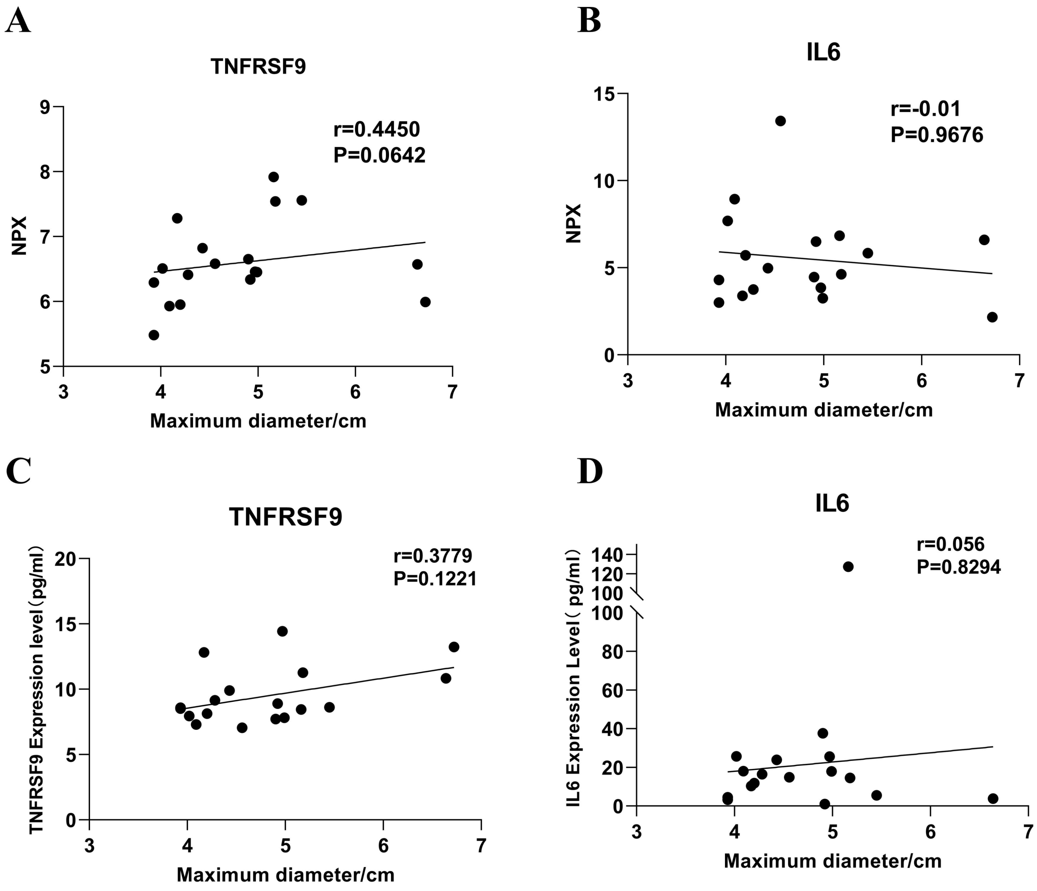

Supplement Figure 2. Correlations between TNFRSF9 and IL6 levels and the maximum diameter in AAA patients. (A) Correlation between the NPX value of TNFRSF9 and the maximum diameter in AAA patients. (B) Correlation between the NPX value of IL6 and the maximum diameter in AAA patients. (C) Correlation between the ELISA value of TNFRSF9 and the maximum diameter in AAA patients. (D) Correlation between the ELISA value of IL6 and the maximum diameter in AAA patients.
